# Impact of Target Oxygenation on the Chemical Track Evolution of Ion and Electron Radiation

**DOI:** 10.3390/ijms21020424

**Published:** 2020-01-09

**Authors:** Daria Boscolo, Michael Krämer, Martina C. Fuss, Marco Durante, Emanuele Scifoni

**Affiliations:** 1 Biophysics Department, GSI Helmholtzzentrum für Schwerionenforschung, 64291 Darmstadt, Germany; m.kraemer@gsi.de (M.K.); m.fuss@gsi.de (M.C.F.); m.durante@gsi.de (M.D.); 2 Institut für Festkörperphysik, TUDarmstadt, 64289 Darmstadt, Germany; 3 Trento Institute for Fundamental Physics and Applications (TIFPA), National Institute for Nuclear Physics, (INFN), 3812 Povo, Italy; emanuele.scifoni@tifpa.infn.it

**Keywords:** radiation track chemistry, chemical track structure, oxygen effect, oxygen depletion, ion beam therapy, ROS, superoxide anion

## Abstract

The radiosensitivity of biological systems is strongly affected by the system oxygenation. On the nanoscopic scale and molecular level, this effect is considered to be strongly related to the indirect damage of radiation. Even though particle track radiolysis has been the object of several studies, still little is known about the nanoscopic impact of target oxygenation on the radical yields. Here we present an extension of the chemical module of the Monte Carlo particle track structure code TRAX, taking into account the presence of dissolved molecular oxygen in the target material. The impact of the target oxygenation level on the chemical track evolution and the yields of all the relevant chemical species are studied in water under different irradiation conditions: different linear energy transfer (LET) values, different oxygenation levels, and different particle types. Especially for low LET radiation, a large production of two highly toxic species (${\mathrm{HO}}_{2}^{•}$ and $O_{2}^{•-}$), which is not produced in anoxic conditions, is predicted and quantified in oxygenated solutions. The remarkable correlation between the ${\mathrm{HO}}_{2}^{•}$ and $O_{2}^{•-}$ production yield and the oxygen enhancement ratio observed in biological systems suggests a direct or indirect involvement of ${\mathrm{HO}}_{2}^{•}$ and $O_{2}^{•-}$ in the oxygen sensitization effect. The results are in agreement with available experimental data and previous computational approaches. An analysis of the oxygen depletion rate in different radiation conditions is also reported. The radiosensitivity of biological systems is strongly affected by the system oxygenation. On the nanoscopic scale and molecular level, this effect is considered to be strongly related to the indirect damage of radiation. Even though particle track radiolysis has been the object of several studies, still little is known about the nanoscopic impact of target oxygenation on the radical yields. Here we present an extension of the chemical module of the Monte Carlo particle track structure code TRAX, taking into account the presence of dissolved molecular oxygen in the target material. The impact of the target oxygenation level on the chemical track evolution and the yields of all the relevant chemical species are studied in water under different irradiation conditions: different linear energy transfer (LET) values, different oxygenation levels, and different particle types. Especially for low LET radiation, a large production of two highly toxic species (${\mathrm{HO}}_{2}^{•}$ and $O_{2}^{•-}$), which is not produced in anoxic conditions, is predicted and quantified in oxygenated solutions. The remarkable correlation between the ${\mathrm{HO}}_{2}^{•}$ and $O_{2}^{•-}$ production yield and the oxygen enhancement ratio observed in biological systems suggests a direct or indirect involvement of ${\mathrm{HO}}_{2}^{•}$ and $O_{2}^{•-}$ in the oxygen sensitization effect. The results are in agreement with available experimental data and previous computational approaches. An analysis of the oxygen depletion rate in different radiation conditions is also reported.

## 1. Introduction

The radiosensitivity of biological systems is strongly affected by the system oxygenation level. Based on evidence from *in vitro* experiments [1,2,3], tissues in hypoxic conditions, or with hypoxic regions, may be up to three times more radioresistant compared to well-oxygenated ones [3]. This effect is one of the main limiting factors for the tumor control in radiotherapy applications, correlating very often with poor prognosis [4] and is generally quantified by the oxygen enhancement ratio (OER). The OER is defined, for a given equal biological effect, as the ratio between the corresponding dose values in fully anoxic and in oxygenated conditions,

(1) $$\mathrm{OER}\left(pO_{2}\right)=\frac{D_{\mathrm{hypoxia}}}{D_{pO_{2}}}{|}_{\mathrm{same}\;\mathrm{effect}}.$$

On the nanoscopic level, the oxygen effect is considered to be strongly related to indirect radiation damage [5] and, in particular, to the ${\mathrm{OH}}^{•}$ radicals [6]. Among the chemical species produced by water radiolysis, ${\mathrm{OH}}^{•}$ radicals are believed to be the most harmful; they have a very short half-life and can react with almost every molecule, including DNA [7]. In oxygenated conditions, the molecular oxygen may react with the damaged molecule stabilizing the damage and making it more difficult to repair [8,9]. Additionally, in oxygenated media, the radiolytic species produced during irradiation can interact with the molecular oxygen dissolved in the target and lead to an enhanced production of highly toxic reactive oxygen species (ROS). Solvated electrons, $e_{\mathrm{aq}}^{-}$, and hydrogen atoms, $H^{•}$ , are generated in large quantities and react to form the superoxide anion, $O_{2}^{•-}$ and its protonated form ${\mathrm{HO}}_{2}^{•}$ , which have been identified as possibly responsible for the oxygen-driven sensitization effect [10].

(2) $$e_{\mathrm{aq}}^{-}+O_{2}\to O_{2}^{•-}$$

(3) $$H^{•}+O_{2}\to {\mathrm{HO}}_{2}^{•}.$$

These species are particularly damaging, since they are involved in the lipid peroxidation chain and play an important role in the production of other toxic species, such as hydroxyl radicals ${\mathrm{OH}}^{•}$ through the Haber-Weiss reaction (catalyzed by the presence of transition metals), peroxynitrite ions ONOO${}^{-}$through the interaction with nitrogen monoxide ${\mathrm{NO}}^{•}$, and $H_{2}O_{2}$ after scavenging by superoxide dismutase enzymes (SOD). The latter theory is supported by both theoretical approaches [11,12,13,14,15] and chemical and biological experimental observations [16,17,18,19] mainly based on studies on the Fenton reaction and on the relation with SOD.

Densely ionizing radiation has been shown to mitigate hypoxia-induced radioresistance [20], motivating a growing interest in ion radiation therapy, especially with high charge, Z, like carbon [21] or better oxygen [22] for the treatment of hypoxic tumors. At the pre-clinical level, new optimization techniques accounting for the oxygenation level and ion linear energy transfer (LET) have recently been developed for particle therapy [3,21].

On the microscopic scale, this effect can be explained as a track density effect. The recombination probability of water-induced free radicals increases with LET, resulting in a lower contribution of the indirect effect of radiation damage and, thus, decreasing the impact of the target oxygenation condition. However, this is not a unique explanation, many other processes might be involved as well and several additional theories have been developed. One of the most accepted theories, the so-called “oxygen in track hypothesis", suggests that the production of $O_{2}$ molecule via multiple ionization processes in the track of densely ionizing radiation can cause locally a partially oxygenated response [23,24,25,26]. Other possible hypotheses are the interacting radical theory [20], the oxygen depletion in the heavy ion tracks [27], the lesion complexity hypothesis [28], and the radical multiplicity [29].

Though many theories have been developed, the nanoscopic processes involved in the oxygenation effect still have to be clarified and very little experimental data at that scale is available. Monte Carlo track structure codes are particularly suitable for studying the microscopic processes involved in the radiation damage. Among them, several codes are able to describe the transport of particle radiation in a medium including the chemical stage of radiation effect. In most of the cases, however, the chemical evolution of a particle track is described in pure water, without taking into account the impact of any dissolved species, like molecular oxygen, on the chemical reaction chain. Recently the Monte Carlo particle track structure code TRAX has been extended to the pre-chemical and chemical stage of radiation in water. A full description of the code can be found in Boscolo et al. [30]. With the new TRAX-CHEM module, the production, diffusion, and interaction of radiation-induced water-derived radicals can be studied with a step-by-step approach under different irradiation conditions.

A further extension of the code, able to account for different concentrations of dissolved molecular oxygen in the target material, is presented in this work. In order to limit the computational costs of the simulation, the dissolved oxygen molecules are assumed to be uniformly distributed in the target material and are treated as a continuum. Time-dependent and LET-dependent yields of all the considered radiolytic species at different oxygenation levels have been studied for different ion radiations and energies.

## 2. Results

### 2.1. Radiolysis of Oxygenated Water

**Figure 1:**
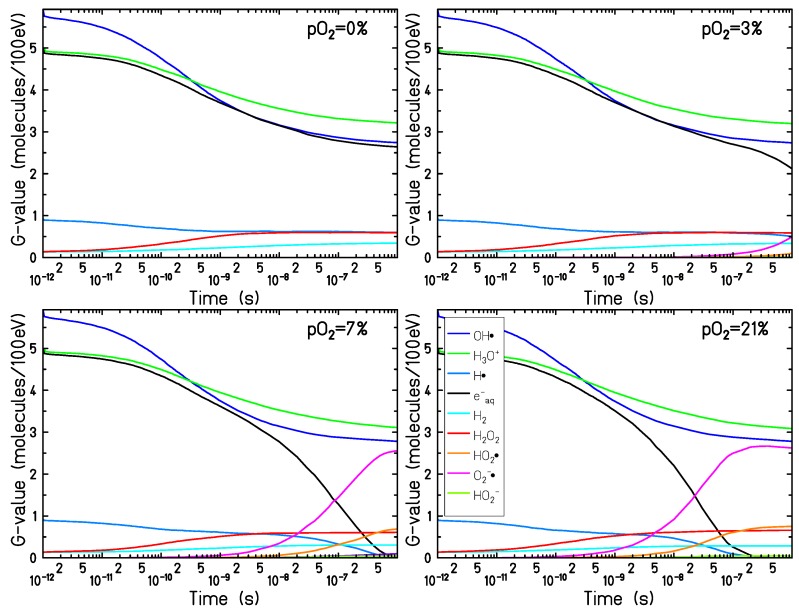
Time dependent yield of the chemical species generated by a 90 MeV proton track (linear energy transfer (LET) = 0.56 keV/μm) in a pure water target (**top left**) and in an oxygenated water target in equilibrium with an atmospheric partial oxygen pressure at the water surface of 3% (**top right**), 7% (**bottom left**), and 21% (**bottom right**).

The time-dependent yield of the chemical species has been evaluated for different target oxygenations and radiation qualities in the time interval $10^{-12}$–$10^{-6}$ s. Figure 1 shows the chemical evolution of 90 MeV proton radiation in targets under four different oxygenation conditions: 0% (complete anoxia), 3% (compatible with typical tumor oxygenation levels), 7% (in the range of normal tissue oxygenation, a condition called “physioxia") and for a target in fully aerated conditions 21%.

In the early stage of the chemical track evolution (up to ∼ 1 ns after the passage of radiation) the radical yields are not affected by the presence of dissolved molecular oxygen in the target and follow the normal water radiolysis behavior. The ion track is very dense and the interaction among the radiation-induced radicals dominates the chemical evolution (independently of the target oxygenation level). At this stage the main products of water radiolysis (${\mathrm{OH}}^{•}$, $H_{3}O^{+}$, $e_{\mathrm{aq}}^{-}$) are the most abundant species; their yield is maximum at the beginning of the chemical stage and decreases with time, as these species are involved in many reaction processes and are consumed during the chemical track evolution. At the same time, the yield of the main reaction products ($H_{2}O_{2}$, $H_{2}$, and ${\mathrm{OH}}^{-}$) increases during the chemical stage.

After the early stage of the chemical track evolution (1 ns after the passage of radiation), the radical distribution becomes more diffuse and the track dynamics become slower. As a consequence, the interaction of the radiolytic species with the dissolved oxygen becomes more prominent and the chemical track dynamics start to depend strongly on the target oxygenation level.

The main effect of the target oxygenation is the consumption of the $e_{\mathrm{aq}}^{-}$ and $H^{•}$ , which are strongly scavenged by the molecular oxygen (Equations (2) and (3)). For $pO_{2}=7\%$ and $pO_{2}=21\%$ a complete consumption of the $e_{\mathrm{aq}}^{-}$ and $H^{•}$ can be observed after 0.8 μs and 0.2 μs, respectively. In the case of $pO_{2}=3\%$, only a small decrease with respect to the completely anoxic target in the electron yield can be observed at the end of the chemical stage. However, on a larger timescale, exceeding the range covered by the TRAX-CHEM code, all these $e_{\mathrm{aq}}^{-}$ and $H^{•}$ will be eventually depleted.

Together with the $e_{\mathrm{aq}}^{-}$ and $H^{•}$ consumption, the production of $O_{2}^{•-}$ and ${\mathrm{HO}}_{2}^{•}$ is the major effect of dissolved oxygen in the target during the process of water radiolysis. The production yield of ${\mathrm{HO}}_{2}^{-}$ is negligible over the time covered by the calculations, for all oxygen concentrations analyzed.

**Figure 2:**
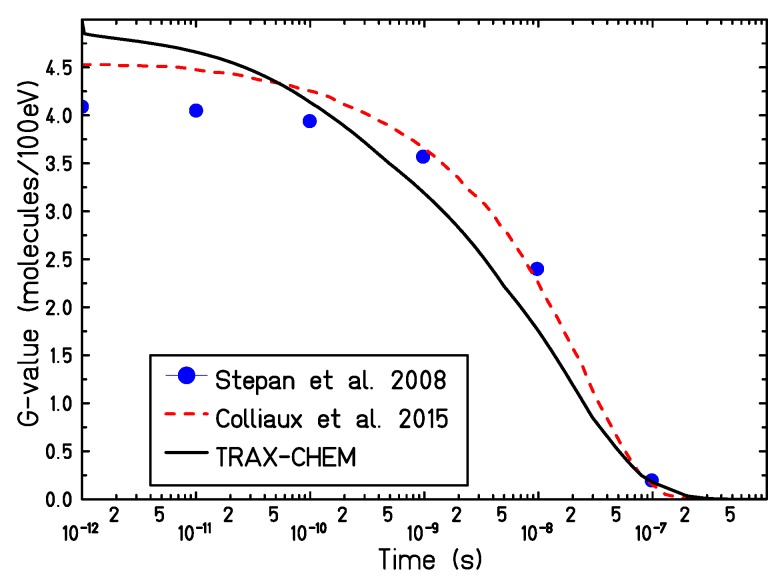
Comparison of different time dependent calculated yield for $e_{\mathrm{aq}}^{-}$ produced by irradiation with 5 MeV protons in a target with a partial oxygen pressure in air of 21%. —— : TRAX-CHEM, –––: Colliaux et al. [15], •: Štepán and Davídková [31].

In Figure 2, the calculated time-dependent yield of solvated electrons, produced by a proton track of 5 MeV, in a target with a partial oxygen pressure in air of 21% has been compared with different chemical track structure codes [15,31]. The initial electron yield, simulated by the TRAX-CHEM code, is higher as compared to the other simulation approaches. This can derive from the use of different cross-section sets or different dissociation models adopted by the different codes [32]. However, due to the lack of experimental data, large variability exists in the predicted radical yield at the very early stages of the chemical evolution [33]. At later stages of the track evolution, all codes show good agreement and predict a full electron consumption at about 0.2 μs after the passage of radiation.

### 2.2. Time-Dependent Radiolytic Yield for Different Oxygen Concentrations

**Figure 3:**
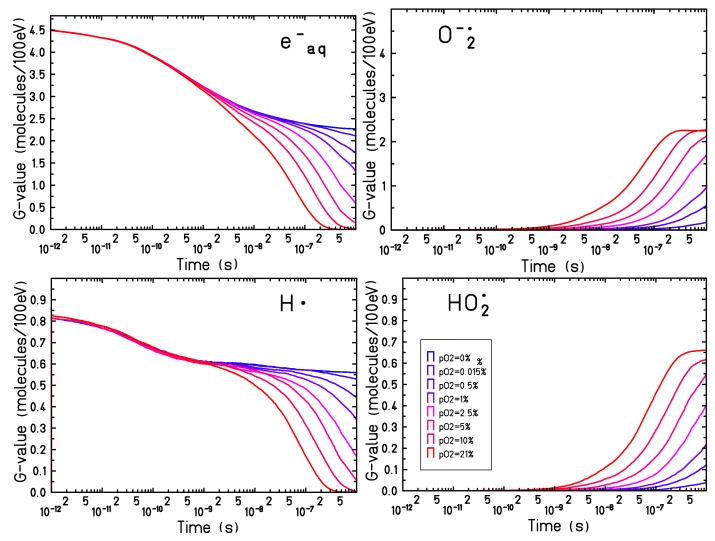
Time dependent yield for $e_{\mathrm{aq}}^{-}$ (**top left**), $H^{•}$ (**bottom left**), $O_{2}^{•-}$ (**top right**) and ${\mathrm{HO}}_{2}^{•}$ (**bottom right**) in an oxygenated water target, with oxygen concentration on the water surface between $pO_{2}=0\%$ and $pO_{2}=21\%$, for 500 keV electron irradiation.

Since the major effects of target oxygenation have been observed on the time evolution of $e_{\mathrm{aq}}^{-}$, $H^{•}$ , ${\mathrm{HO}}_{2}^{•}$ and $O_{2}^{•-}$, their time-dependent yields have been studied for a set of oxygen concentrations ranging between $pO_{2}=0\%$ and $pO_{2}=21\%$. Figure 3 shows the simulation results for 500 keV electron tracks.

An increasing production of $O_{2}^{•-}$ and ${\mathrm{HO}}_{2}^{•}$ (and a decrease in the yield of $e_{\mathrm{aq}}^{-}$ and $H^{•}$ ) is observed for all oxygen concentrations. The oxygen reaction dynamics become faster when increasing the oxygen concentration and, for oxygenation levels above $pO_{2}=5\%$, complete consumption of the $e_{\mathrm{aq}}^{-}$ and $H^{•}$ can be observed within a microsecond, leading to a saturation in the production of $O_{2}^{•-}$ and $H_{2}O$ . The yield at the saturation level of the $O_{2}^{•-}$nearly matches the yield of the solvated electrons in anoxic conditions: in fully oxygenated conditions ($pO_{2}=21\%$) the yield of $O_{2}^{•-}$ $G_{O_{2}^{-}}\left(10^{-6}s,pO_{2}=21\%\right)=2.24$ and the electron yield in anoxia is $G_{e_{\mathrm{aq}}^{-}}\left(10^{-6}s,pO_{2}=0\%\right)=2.25$.

The production yield of the ${\mathrm{HO}}_{2}^{•}$ is slightly larger compared to the maximum yield of the $H^{•}$ in hypoxic conditions: $G_{{\mathrm{HO}}_{2}^{•}}\left(10^{-6}s,pO_{2}=21\%\right)=0.66$ while $G_{H^{•}}\left(10^{-6}s,pO_{2}=21\%\right)=0.56$. Even though the larger part of the ${\mathrm{HO}}_{2}^{•}$ is produced through the reaction process described in Equation (3), a smaller but not negligible contribution of the ${\mathrm{HO}}_{2}^{•}$ yield comes from the recombination of $O_{2}^{•-}$ with $H_{3}O^{+}$, (reaction (xxiii) in Table 1):

(4) $$H_{3}O^{+}+O_{2}^{•-}\to {\mathrm{HO}}_{2}^{•}.$$

In the present simulations, pH and the acid-base equilibrium of ${\mathrm{HO}}_{2}^{•}$ and $O_{2}^{•-}$ are not modeled explicitly, so that all $G_{O}2^{-}$ and $G_{\mathrm{HO}}2^{•}$ reflect their production by radiolysis rather than a stable concentration. The $pK_{a}$ of 4.8 leads to an equilibrium ratio [$O_{2}^{•-}$]/[${\mathrm{HO}}_{2}^{•}$] at neutral pH of about 250. Accordingly with what is observed in Figure 1, for targets at oxygenation levels larger than 5% a complete consumption of $e_{\mathrm{aq}}^{-}$ and $H^{•}$ can be observed and the chemical evolution reaches an equilibrium. For less oxygenated targets, however, the chemical dynamic is slower and the equilibrium is not reached within a microsecond and can proceed in a complex way at larger timescales.

### 2.3. $pO_{2}$-Dependent Radiolytic Yield for Different LET

**Figure 4:**
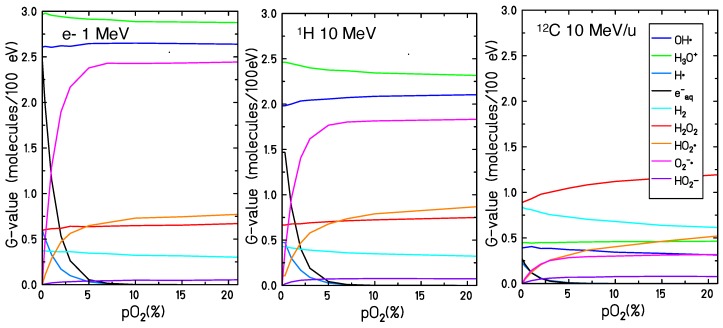
Radiolytic yields, at 1 μs, for all the different chemical species generated by the water radiolysis at different oxygenation conditions by 1 MeV electron irradiation (LET= 0.13 keV/μm) on the left panel and 10 MeV proton (LET= 3.9 keV/μm) in the central panel and 10 MeV/u carbon ions radiation (LET= 133 keV/μm) on the right panel.

The final radical yield (t = 1 μs, i.e., at the very end of the chemical stage) of all chemical species has been studied as a function of the oxygen concentration. Figure 4 shows the results of the calculations performed for different particles and different LET values: an electron track of 1 MeV, a proton track of 10 MeV and a carbon track of 10 MeV/u. The total yield of the chemical species is larger for low LET radiation and decreases when increasing the LET. For high LET radiation, the reaction kinetics is much faster: the ion track is denser, resulting in a larger recombination probability of the chemical species generated during the water radiolysis. Additionally, the yield of the recombination products ($H_{2}O_{2}$, $H_{2}$ and ${\mathrm{HO}}_{2}^{•}$ ) increases when increasing the LET.

The general trend of the radical yield at the different oxygenation conditions is similar for all the radiation qualities investigated. In all cases, the chemical species affected most by the dissolved molecular oxygen are $O_{2}^{•-}$, ${\mathrm{HO}}_{2}^{•}$, $e_{\mathrm{aq}}^{-}$ and $H^{•}$ . A rapid decrease in the yield of the $e_{\mathrm{aq}}^{-}$ and $H^{•}$ with increasing target oxygenation level can be observed up to $pO_{2}∼5\%$. For larger oxygenation levels, $e_{\mathrm{aq}}^{-}$ and $H^{•}$ are completely depleted. Accordingly, a steep increase of the production yield of $O_{2}^{•-}$ and ${\mathrm{HO}}_{2}^{•}$ is observed for oxygen concentrations up to $pO_{2}∼5\%$, but further increasing this value a saturation level is reached for the $O_{2}^{•-}$ while the yield of the ${\mathrm{HO}}_{2}^{•}$ continues to increase but in a much slower way.

The production yield of all the other radiolytic species is less significantly modified by the water oxygenation level. An increase of the $H_{2}O_{2}$ yield with the oxygen concentration can be observed especially for higher LET radiation. For 10 MeV/u carbon ions, the G-value at 1 μs of the $H_{2}O_{2}$ increases from 0.89 (for the anoxic case, $pO_{2}=0\%$) to 1.19 (for the fully oxygenated target, $pO_{2}=21\%$). For the 1 MeV electron radiation the $H_{2}O_{2}$ G-value goes from 0.6 (in the anoxic target) to 0.67 (in the target with $pO_{2}=21\%$). The time dependent yield of the $H_{2}O_{2}$ is the result of two main processes:

(5) $${\mathrm{OH}}^{•}+{\mathrm{OH}}^{•}⟶H_{2}O_{2}$$

(6) $$e_{\mathrm{aq}}^{-}+H_{2}O_{2}⟶{\mathrm{OH}}^{•}+{\mathrm{OH}}^{-}.$$

The first process is dominant at the early stages of the chemical track evolution and is the main production channel of the $H_{2}O_{2}$ while the second process becomes significant after 1 ns and removes $H_{2}O_{2}$ from the target. The contribution of the first process is more relevant in the dense primary radical condition after, high LET radiation, and is potentiated by the absence of the second one if sufficient molecular oxygen is present (due to competition with the molecular oxygen scavenging effect). The combined effect results in a larger yield of $H_{2}O_{2}$ at 1 μs after irradiation. A small increase of the ${\mathrm{OH}}^{•}$ radical yield and a small decrease of the $H_{3}O^{+}$, $H_{2}$ and ${\mathrm{OH}}^{-}$ yield can be observed, but in these cases, changes in the production yield at the microsecond are lower than 10%.

**Figure 5:**
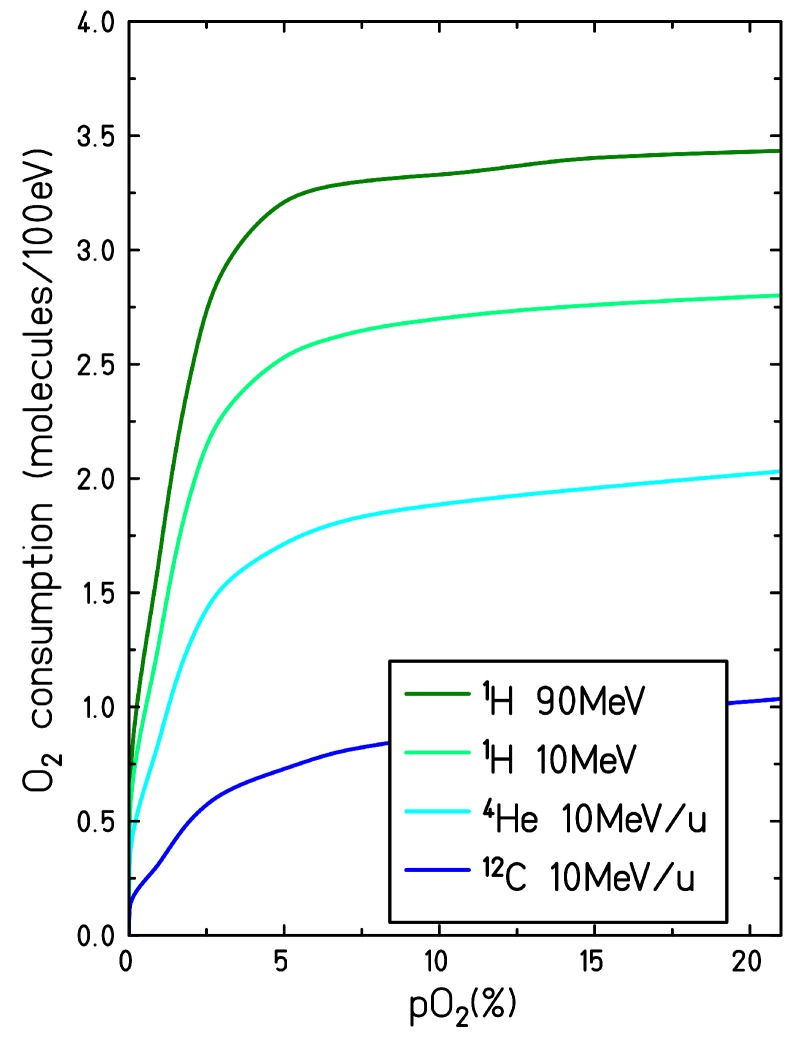
Consumption of the molecular oxygen for different target oxygenation levels and induced by different LET radiations. The consumption yields are calculated at the end of the chemical track evolution, t = 1 μs.

Figure 5 represents the consumption yield of the molecular oxygen at the end of the chemical stage, as a function of the target oxygenation, for different ion radiation qualities: 90 MeV proton (LET = 0.56 keV/μm), 10 MeV proton (LET = 3.9 eV/μm)), 10 MeV/u helium ion (LET = 15.2 keV/μm) and 10 MeV/u carbon ion (LET = 133 keV/μm). The formation of molecular oxygen through second-order recombination processes (see reactions (xvi), (xvii), (xxv) and (xxvi) in Table 1) is also taken into account. In fully oxygenated conditions and for high LET radiation tracks, at 1 μs after the passage of radiation, up to 25% of all the molecular oxygen initially depleted is regenerated in the target through these secondary processes.

The “yield” of oxygen consumption increases with increasing target oxygenation until reaching a plateau at $pO_{2}=5\%$ when all the radiation-induced solvated electrons are scavenged. Though the plateau starts at $pO_{2}=5\%$ for all the radiation quality investigated, the total yield of oxygen consumption is higher for low LET radiation. In contrast, for high LET radiation, the maximum yield of oxygen consumed is lower and the decrease at low oxygenation is more moderate.

### 2.4. Radiolytic Yields for Different LET and Particle Type in Oxygenated Water

**Figure 6:**
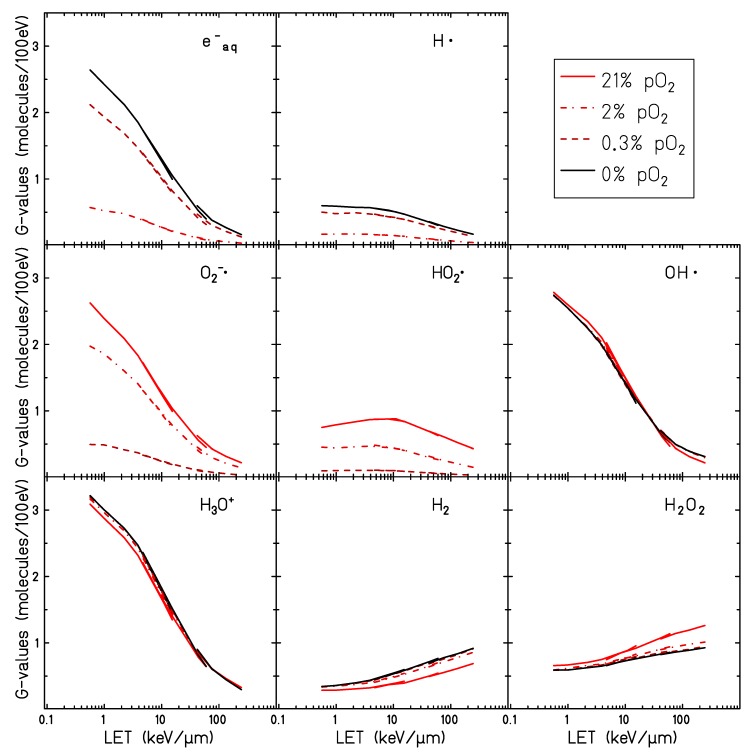
LET dependent radiolytic yields for all chemical species generated by the water radiolysis in water target at a partial oxygen pressure of 0%, 0.3%, 2% and 21%. Calculations were performed with protons, helium ions and carbon ions at one microsecond after the irradiation.

The impact of dissolved molecular oxygen on the final radical production yield has been studied under different oxygen concentrations for different particle radiation and different energies. Water targets at oxygenation levels of 21%,3%,0.2% and 0%, respectively, irradiated by protons, helium, and carbon ions with LET values ranging between 0.14 and 232 keV/μm are investigated here. LET dependent yields at the completion of the chemical stage are shown in Figure 6.

These results are in agreement with what has been already observed in Figure 3 and Figure 4: the solvated electrons and the atomic hydrogen ($e_{\mathrm{aq}}^{-}$ and $H^{•}$ ) yields decrease significantly with the increase of the oxygen concentration until full depletion of these species is observed in the case of complete oxygenation for all the radiation qualities investigated. The yields of $O_{2}^{•-}$ and ${\mathrm{HO}}_{2}^{•}$, which are the two main indicators of the presence of molecular oxygen in the target, increase when increasing the target oxygenation over the entire range of LETs investigated. Their production yield is maximum for lower LET radiation and decreases for higher LET.

Only minor effects of the target oxygenation are observed for the other chemical species generated by water radiolysis. The scavenging effect of the solvated electrons and atomic hydrogen radicals leads to a general decrease in the production of the $H_{2}$ molecule which is mainly generated as a product of the recombination processes described by reactions (vi) and (x) in Table 1:

(7) $$e_{\mathrm{aq}}^{-}+e_{\mathrm{aq}}^{-}+2H_{2}O⟶H_{2}+2{\mathrm{OH}}^{-}$$

(8) $$H^{•}+H^{•}⟶H_{2}.$$

Consistent with what is shown in Figure 4, increased production of $H_{2}O_{2}$ can be observed in oxygenated conditions at high LET, while no effect is apparent at low LET.

The yield of the ${\mathrm{OH}}^{•}$ radical is slightly higher in an oxygenated target for low LET, while at intermediate LET no difference between oxygenated and hypoxic target is observed. A larger yield of ${\mathrm{OH}}^{•}$ is observed in the anoxic case in the high LET region. One of the main processes consuming the ${\mathrm{OH}}^{•}$ radical is its interaction with a solvated electron. For oxygenated targets, this reaction is directly competing with the interaction of the $e_{\mathrm{aq}}^{-}$ with the $O_{2}^{•-}$ and results in a lower amount of scavenged ${\mathrm{OH}}^{•}$. For high LET, however, the track kinetic is faster and the ${\mathrm{OH}}^{•}$ reacts with the $e_{\mathrm{aq}}^{-}$ before the interactions with the dissolved oxygen become dominant.

The discontinuities are shown on the LET dependent curves in Figure 6 are due to the different simulated radiation types. This is because the LET is not a unique parameter for describing a particle track structure, and it also depends on the charge and speed of the primary particle. However, the dependence on the particle seems to vary not significantly with the oxygenation level of the target.

## 3. Discussion

Motivated by the need for a better understanding of the nanoscopic processes underlying the oxygen-induced radiosensitivity, the chemical track dynamics of the radiolytical species generated by different radiation qualities has been studied for water targets at different oxygenation levels.

Time-dependent radical yields for targets at different oxygenation levels have been calculated for proton and electron radiation, Figure 1 and Figure 3. For all the investigated conditions, the impact of the target oxygenation can be observed only in the later stages of the chemical track evolution, indicating that for the first nanoseconds the radical yields are determined only by the intra-track recombination processes, independent from the target conditions.

**Figure 7:**
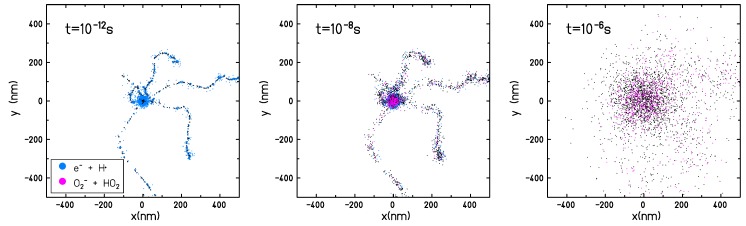
Chemical evolution of a 10 MeV/u carbon ion track in an oxygenated water target with an oxygen partial pressure $pO_{2}=21\%$. Track shown in beam eye view.

The main effect of the dissolved molecular oxygen in the target is the consumption of $e_{\mathrm{aq}}^{-}$ and $H^{•}$ and a corresponding production of $O_{2}^{•-}$ and ${\mathrm{HO}}_{2}^{•}$ (see reactions (2) and (3)). At 1 μs after the passage of radiation, a complete depletion of $e_{\mathrm{aq}}^{-}$ and $H^{•}$ is observed for oxygen concentrations larger than $pO_{2}=5\%$. For oxygen concentrations lower than $pO_{2}=5\%$ the probability of interacting with the dissolved molecular oxygen is lower and the equilibrium on the radical yields is not reached within a microsecond (the time frame covered by the TRAX-CHEM simulations, and normally considered as the end of the chemical stage). The temporal interval of the simulation is chosen in a way that the chemical track evolution can be considered concluded; the radical distribution can be assumed to be uniform and the reaction process is determined only by the reactant concentration and not by their spatial distribution. As shown in Figure 7 the track structure is lost on the microsecond time scale and only a slightly increased radical concentration can be observed on the micrometer scale close to the track center. At the conclusion of the chemical stage, in completely anoxic conditions, when only the intra-track reactions are accounted for, the reaction rates of the different radicals are very low and the yields of the different species become constant. Typical proton and electron track radii corresponding to the end of the chemical stage are in the order of several hundreds of nanometer up to one micrometer.

However, when interactions with target molecules are possible, such as in oxygenated conditions, the radiolytic species will keep interacting with the target even after the track structure is completely lost. The radical yields will not reach equilibrium within the μs time frame and the chemical kinetics can proceed in a complex way for a very long time [15]. In the case of oxygenated water, according to our model (Table 1), the only species able to interact with the dissolved oxygen are $e_{\mathrm{aq}}^{-}$ and $H^{•}$ . Thus, it is to be expected that the whole track reaction kinetics will be limited to the lifetime of these two species in the target material. The chemical evolution of homogeneous systems is beyond the scope of this study; therefore, it has been decided to not extend the simulation time but to limit the study to the accepted time frame of the track evolution.

The radical production yields at the completion of the chemical track evolution have been studied for different radiation types and oxygenation levels. In Figure 4 the G-values for all the radiolytic species as a function of the oxygen concentration are studied for 1 MeV electron, 10 MeV proton and 10 MeV/u carbon ion radiation, while in Figure 6 the LET dependence of the radical production yield is reported for 21%, 2%, 0.3% and 0% $pO_{2}$. The yield of $O_{2}^{•-}$ and ${\mathrm{HO}}_{2}^{•}$ increases with increasing target oxygenation over the whole range of analyzed LET. Their production yield is maximum for lower LET radiation and decreases for higher LET values. This strong dependence on the LET can be explained as a track structure effect: for high LET the ion track is denser and radicals are produced in close proximity. The radiation-induced water radicals will, then, recombine reacting with each other before any significant oxygen scavenging effect. Similar results have been obtained by Colliaux et al. [15] where the LET dependent yield of (${\mathrm{HO}}_{2}^{•}$ + $O_{2}^{•-}$ ) has been calculated in an oxygenated water target with $pO_{2}=21\%$ and, as in our calculations, a pronounced decrease in the yield of ($O_{2}^{•-}$ + ${\mathrm{HO}}_{2}^{•}$ ) with LET has been observed. A significant increase in the production of $H_{2}O_{2}$ has been also observed for oxygenated targets, especially for high LET irradiation.

**Figure 8:**
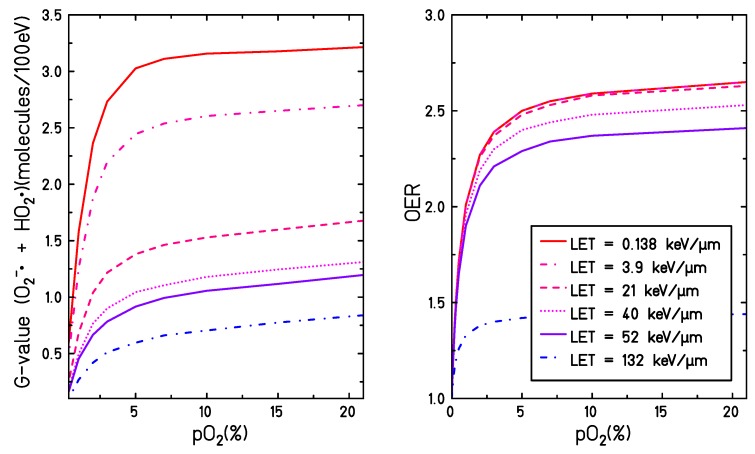
G-values for the production of superoxide and perhydroxyl (**left**) and oxygen enhancement ratio (OER) (**right**) are depicted as a function of target oxygenation. The OER values are calculated according to the parametrization proposed by Tinganelli et al. [3] but using definition (1).

When considering the correlation between the production yield of $O_{2}^{•-}$ and ${\mathrm{HO}}_{2}^{•}$ at different LETs, particle types, and dissolved oxygen concentrations, it is reasonable to believe that the interaction of $e_{\mathrm{aq}}^{-}$ and $H^{•}$ with the molecular oxygen leads directly or indirectly to the production of toxic species, able to damage the cell structure or alter cell signaling. This theory is supported by many studies [11,12,13,14,15,16,17,18,19,34,35,36] and correlates well with *in vitro* experiments, showing that the oxygen enhancement effect in biological systems has a pronounced dependence on the radiation LET [3,37,38] (it decreases when irradiating with higher LET radiation). This parallelism between the production of $O_{2}^{•-}$ and ${\mathrm{HO}}_{2}^{•}$ and the oxygen effects observed in biological systems becomes even more evident when comparing production yields of $O_{2}^{•-}$ and ${\mathrm{HO}}_{2}^{•}$ and the OER curve under different irradiation and target conditions. As shown in Figure 8, the general trend of the OER and of G($O_{2}^{•-}$ +$H_{2}O$ ) as a function of the target oxygenation level are very similar: a steep increase in both curves can be observed when increasing the target oxygenation until reaching a plateau for partial oxygen pressures larger than 5%. Additionally, a reduction of the entire OER curve and G($O_{2}^{•-}$ +$H_{2}O$ ) is observed when increasing the LET for all the oxygenation levels. However, the OER curve shows a maximum sensitivity on the LET for values ∼ 100 keV/μm while the radical yield has a maximum sensitivity for LET values ∼ 10 keV/μm. Therefore, it is not straightforward to deduce the oxygenation effect in biological systems directly within the present theoretical framework. The present study is focused, indeed, on assessing the role of one possible sensitization mechanism but additional pathways, e.g., the oxygen fixation, must be also taken into account when aiming at a complete explanation of the oxygen-induced radiation sensitivity. Additionally, a water target is a considerably simplified system compared to the cellular environment and all the complex reaction chains taking place with cell medium, including the secondary reactions taking place at further stages, the biological damage and its repair, and the possible cross-talk with signaling pathways caused by altered levels of some ROS, are not accounted for. In this context, further extensions of the model can be considered in order to take into account the presence of additional solutes known to play an important role in the induction of radical damage, such as nitrogen monoxide ${\mathrm{NO}}^{•}$, carbonate or bicarbonate ions, or the presence of metals to catalyze the Fenton chemistry [34,39]. At the same time, radical scavengers such as superoxide dismutase (SOD), catalase (CAT), gluthatione peroxidase (GSH) could be included in the model [40]. However, considering that all these species will only have a role in the system dynamic at later stages of the chemical track evolution, when the primary radiation-induced radicals are already diffused, computationally lighter approaches based on the homogeneous chemistry might be considered appropriate for the implementation of further stages of the system dynamics.

The investigation of the influence of target oxygenation and LET on radiation-induced radical production and oxygen consumption opens the way for applications where these factors are being discussed to enable a differential radioprotective effect. This includes e.g., ultra-high dose rate (FLASH) conditions [39,41,42] where high instantaneous concentrations of ROS are produced and replenishment of oxygen through diffusion is too slow to maintain stable oxygenation.

## 4. Materials and Methods

### 4.1. Simulation of Particle Track Evolution in Non-Oxygenated Water

The evolution of charged particle tracks is described in TRAX-CHEM as a three steps process: the physical, the pre-chemical and the chemical stage. These three stages take place subsequently and are characterized by a characteristic time scale. The physical stage of the radiation track evolution consists of the simulation of the ionization and excitation processes along the particle track and is simulated with the standard version of the TRAX code [43]. The electron and ion tracks are simulated with an event-by-event approach, until they reach a cutoff energy of 7.4 eV, which corresponds to the lower electronic excitation level of water. Ion and electron interactions are described through a set of shell specific ionization and excitation cross-section tables, and Auger electron production and electron elastic scattering cross sections are implemented as well. Multiple ionization processes and photon interactions are not implemented in the current version of the TRAX code. At the conclusion of this stage, which is assumed to be at $10^{-15}s$ after the irradiation, the positions and the shell specific ionization or excitation levels of the target water molecules are provided and can be used as an input for the following stage.

The pre-chemical stage, which lasts up to $10^{-12}s$, consists of the dissociation and thermalization of all the products generated during the physical stage: ${\mathrm{HO}}_{2}^{+}$, ${\mathrm{HO}}_{2}^{*}$ and $e_{\mathrm{aq}}^{-}$. The probability of undergoing a dissociation process or relaxing to the ground state depends on the specific ionization or excitation channel. In TRAX-CHEM, all the ionized molecules are thought to dissociate to ${\mathrm{OH}}^{•}$ and $H_{3}O^{+}$, while four possible dissociation patterns have been considered for excited water molecules: auto-ionization, two dissociative decays ( ${\mathrm{OH}}^{•}$ + $H^{•}$ and $H_{2}$ + $H_{2}O_{2}$) and relaxation to the ground state. During the dissociation process, a fraction of energy is transferred to the dissociation fragments as kinetic energy. These species will then need to release this energy and thermalize with the surrounding medium before starting to behave and interact as chemical species. A complete description of the dissociation and thermalization model has been reported in Boscolo et al. [30]. At the end of the thermalization process, the pre-chemical stage is considered to be concluded and the last and longest stage of the track evolution begins the chemical stage.

During the chemical stage, the radiolytic species diffuse and interact among themselves until reaching the chemical equilibrium. In TRAX-CHEM these two processes are described with a step by step approach, which allows us to determine the position of each chemical species in every step of the simulation. For every time step, the Brownian diffusion process is modeled with a jump in a random direction. The reaction model is described through a proximity parameter; the reaction radius. If two species are closer than the corresponding reaction radius, the reaction is supposed to take place: the two reactants are removed from the chemical list and substituted by the reaction products. Inter-track reactions (i.e., reactions of chemical species originating from different primary particles) are not included in the present TRAX-CHEM extension. Details on the implementation of the chemical model including the stepping algorithm, the reaction models, the calculation of the reaction radii and the diffusion model have been presented in Boscolo et al. [30]. Complete lists of the reactions and the chemical species implemented in TRAX-CHEM are provided in Table 1 and Table 2; these tables have been updated and extended with respect to the initial ones [30] in order to take into account the presence of dissolved molecular oxygen in the target, as presented in the next section. In TRAX-CHEM the chemical stage, and thus the track evolution, is supposed to conclude $10^{-6}$ s after the physical irradiation interactions. After this time, the chemical yields of the different species become constant and the track development can be considered to be finished.

**Table 1:** List of all the reactions and reaction rate constants, k, used in this work (obtained mainly from [44]). Reactions (14)-(26) arise as a consequence of the presence of dissolved $O_{2}$.

		Reaction		Products	$k(10^{10}{\mathrm{dm}}^{3}{\mathrm{mol}}^{-1}s^{-1})$
	(i)	${\mathrm{OH}}^{•}$+ ${\mathrm{OH}}^{•}$	⟶	$H_{2}O_{2}$	0.6
	(ii)	${\mathrm{OH}}^{•}$+ $e_{\mathrm{aq}}^{-}$	⟶	${\mathrm{OH}}^{-}$	2.2
	(iii)	${\mathrm{OH}}^{•}$+ $H^{•}$	⟶	$H_{2}O$	2.0
	(iv)	${\mathrm{OH}}^{•}$+ $H_{2}$	⟶	$H^{•}$ + $H_{2}O$	0.0045
	(v)	${\mathrm{OH}}^{•}$+ $H_{2}O_{2}$	⟶	${\mathrm{HO}}_{2}^{•}$+ $H_{2}O$	0.0023
	(vi)	$e_{\mathrm{aq}}^{-}$+ $e_{\mathrm{aq}}^{-}$+ $H_{2}O$+ $H_{2}O$	⟶	$H_{2}$+ ${\mathrm{OH}}^{-}$+ ${\mathrm{OH}}^{-}$	0.55
	(vii)	$e_{\mathrm{aq}}^{-}$+ $H^{•}$ + $H_{2}O$	⟶	$H_{2}$+ ${\mathrm{OH}}^{-}$	2.5
	(viii)	$e_{\mathrm{aq}}^{-}$+ $H_{3}O^{+}$	⟶	$H^{•}$ + $H_{2}O$	1.7
	(ix)	$e_{\mathrm{aq}}^{-}$+ $H_{2}O_{2}$	⟶	${\mathrm{OH}}^{•}$+ ${\mathrm{OH}}^{-}$	1.0
	(x)	$H^{•}$ + $H^{•}$	⟶	$H_{2}$	1.0
	(xi)	$H^{•}$ + $H_{2}O_{2}$	⟶	${\mathrm{OH}}^{•}$+ $H_{2}O$	0.01
	(xii)	$H^{•}$ + ${\mathrm{OH}}^{-}$	⟶	$e_{\mathrm{aq}}^{-}$+ $H_{2}O$	0.002
	(xiii)	$H_{3}O^{+}$+ ${\mathrm{OH}}^{-}$	⟶	$H_{2}O$+ $H_{2}O$	10.0
	(xiv)	$e_{\mathrm{aq}}^{-}$+ $O_{2}$	⟶	$O_{2}^{•-}$	1.9
	(xv)	$H^{•}$ + $O_{2}$	⟶	${\mathrm{HO}}_{2}^{•}$	2.0
	(xvi)	${\mathrm{OH}}^{•}$+ ${\mathrm{HO}}_{2}^{•}$	⟶	$O_{2}$	1.0
	(xvii)	${\mathrm{OH}}^{•}$+ $O_{2}^{•-}$	⟶	$O_{2}$+ ${\mathrm{OH}}^{-}$	0.9
	(xviii)	${\mathrm{OH}}^{•}$+ ${\mathrm{HO}}_{2}^{-}$	⟶	${\mathrm{HO}}_{2}^{•}$+ ${\mathrm{OH}}^{-}$	0.5
	(xix)	$e_{\mathrm{aq}}^{-}$+ ${\mathrm{HO}}_{2}^{•}$	⟶	${\mathrm{HO}}_{2}^{-}$	2.0
	(xx)	$e_{\mathrm{aq}}^{-}$+ $O_{2}^{•-}$	⟶	${\mathrm{OH}}^{-}$+ ${\mathrm{HO}}_{2}^{-}$	1.3
	(xxi)	$H^{•}$ + ${\mathrm{HO}}_{2}^{•}$	⟶	$H_{2}O_{2}$	2.0
	(xxii)	$H^{•}$ + $O_{2}^{•-}$	⟶	${\mathrm{HO}}_{2}^{-}$	2.0
	(xxiii)	$H_{3}O^{+}$+ $O_{2}^{•-}$	⟶	${\mathrm{HO}}_{2}^{•}$	3
	(xxiv)	$H_{3}O^{+}$+ ${\mathrm{HO}}_{2}^{-}$	⟶	$H_{2}O_{2}$	2.0
	(xxv)	${\mathrm{HO}}_{2}^{•}$+ ${\mathrm{HO}}_{2}^{•}$	⟶	$H_{2}O_{2}$+ $O_{2}$	0.000076
	(xxvi)	${\mathrm{HO}}_{2}^{•}$+ $O_{2}^{•-}$	⟶	$O_{2}$+ ${\mathrm{HO}}_{2}^{-}$	0.0085

### 4.2. Simulation of Particle Track Evolution in Oxygenated Water

The classical version of the TRAX-CHEM code has been modified and is now able to simulate the chemical evolution of ion tracks in water targets under different oxygen pressure conditions. The particle list and the reaction network of the classical version of the TRAX-CHEM code have been, thus, extended (see Table 1 and Table 2) and new species, generated by the interaction of $O_{2}$ with the radiation-induced water-free radicals, are now included in the track chemical evolution.

In contrast to all other species, which are explicitly included in the code and treated with the step by step approach mentioned above, the molecular oxygen is assumed to be homogeneously distributed in the target and is treated as a continuum. This approximation, proposed by Pimblott et al. [45], Green et al. [46], is necessary to limit the computational cost of the simulations and has also been adopted by other authors [13,14,47,48]. The explicit introduction of the oxygen in the simulation would dramatically increase the computing time, even for very dilute solutions [45]. As an example, in a chemical simulation of a 10 MeV proton track in a cubic volume of 5 μm side the number of radiolytic species produced per particle track is about 10${}^{3}$. In a fully oxygenated condition (21% $pO_{2}$, corresponding to a concentration of 0.27 mmol/litre) the number of oxygen molecule that have to be explicitly introduced (in the same cubic volume of 5μm side) and followed at every single step of the simulation is about 2 × 10${}^{7}$, increasing the simulation time by more than four orders of magnitude. Considering the relatively low radiation-induced oxygen consumption compared to the total amount of molecular oxygen dissolved in the target, a variation of the global oxygen concentration during the track evolution can be excluded for all the oxygenations and radiation conditions examined. When investigating the possibility of noticeable local oxygen depletion in the track cores, it has to be kept in mind that the interaction of the radiolytic species with the target occurs in a later stage of the expanding chemical track evolution since the reaction dynamic between the induced chemical species is slower. For high LET ions (∼100 keV/μm), the highest local density of oxygen removal is reached around 10 ns within 10-20 nm of the track core and stays below 250 μM, quickly decreasing as the chemical track diffuses and oxygen conversion to superoxide and perhydroxyl becomes important. For even larger LET values, only a slightly delayed onset of the ${\mathrm{HO}}_{2}^{•}$ and $O_{2}^{•-}$ production can, therefore, be expected. Similar conclusions have been reported by Colliaux et al. [15]. Additionally, the molecular oxygen production through multiple ionizations is not accounted for here, but it has been demonstrated that the contribution of this process to the radical yield is very low [15].

Under these conditions, given an oxygen concentration $c_{s}$, the probability for a radiolytic species to interact with an oxygen molecule of the target is determined by the rate equation:

(9) $$\frac{d\Omega (t)}{dt}=-k\left(t\right)c_{s}\Omega \left(t\right),$$

where Ω(t) is the time-dependent survival probability of the molecule of interest. The time dependent rate coefficient k(t) for the reaction of interest can be calculated according to the Noyes theory [49], as:

(10) $$k\left(t\right)=4\pi D^{\prime }R_{reac}\left(1+\frac{R_{reac}}{\sqrt{\pi D^{\prime }t}}\right),$$

where $D^{\prime }$ is the relative diffusion coefficient defined, considering the two species A and B, $D^{\prime }=D_{A}+D_{B}$ and $R_{reac}$ is the reaction radius defined according to the Smoluchowski theory, as

(11) $$R_{reac}=\frac{k_{AB}}{4\pi (D_{A}+D_{B})}.$$

The probability for a molecule to react with the dissolved oxygen in a time *t* will, thus, be:

(12) $$W\left(t\right)=1-\Omega \left(t\right)=1-e^{-4\pi D^{\prime }R_{reac}c_{s}\left(t+2R_{reac}\sqrt{\frac{t}{\pi D^{\prime }}}\right)}.$$

Since TRAX-CHEM uses a variable time step, the probability W(t) that a species will interact is calculated for each time step and is sampled through a uniformly distributed random variable x∈[0:1]. When x≤W(t) the reaction is taking place, the reactants are removed from the simulation and replaced by the corresponding reaction products.

### 4.3. Calculation

In order to identify a range of oxygen concentrations compatible with the one of a biological system, the concentration of molecular oxygen dissolved in the water target is calculated from the partial pressure of oxygen in the air within standard conditions of pressure by Henry’s low. Given a gas partial pressure, $p_{gas}$ (in atmospheres), the solubility of the gas at a fixed temperature in a particular solvent, $c_{s}$ (in mol per liter):

(13) $$c_{s}=K_{H}\cdot P_{gas},$$

where $K_{H}$ is Henry’s constant and corresponds to the gas solubility in water. For $O_{2}$ at a temperature of $20{\;}^{\circ }C$, Henry’s constant is $K_{H}=1.3\times 10^{-3}\;\mathrm{mol}/\left(l\;\mathrm{atm}\right)$. In fully oxygenated conditions (partial oxygen pressure $p_{O_{2}}=21\%$), the oxygen concentration equals 0.27mmol/L.

For the results presented in the present simulation results, G values, i.e., numbers of species produced per 100 eV energy deposition, have been calculated for all the chemical species in a simulated volume of 5×5×5 μ$m^{3}$ for low LET radiation. In the case of high LET radiation, a geometry reduced in the beam direction of 5×5×0.5 μ$m^{3}$ was chosen in order to guarantee the track segment condition. For each simulation, a series of parallel calculations were performed in order to reduce the statistical uncertainty. The number of primary particles was chosen so that a total energy of 2.5MeV was deposited in the target. The statistical fluctuation in the chemical simulations has been evaluated for every single primary particle and was shown to be within 10% [30].

## 5. Conclusions

In the presented work, the impact of target oxygenation on radical production yields has been studied for electron and ion radiation in water with the recently implemented extension of the TRAX-CHEM code, able to handle the presence of dissolved molecular oxygen in the target. The molecular oxygen concentrations investigated in this work range from anoxic conditions ($pO_{2}=0\%$) to air-saturated water targets ($pO_{2}=21\%$). Time-dependent and LET dependent yields of all the simulated radiolytic species at different oxygenation levels have been studied for different ion radiation and different energies up to 1 μs after the passage of radiation. In oxygenated conditions, a large production of two highly toxic species ($O_{2}^{•-}$ and ${\mathrm{HO}}_{2}^{•}$) has been predicted, especially for low LET radiation. These species are generated as reaction products, from the interaction of respectively $e_{\mathrm{aq}}^{-}$ and $H^{•}$ with the dissolved molecular oxygen. Thus, a decrease in the final (1 μs) yield of the $e_{\mathrm{aq}}^{-}$ and $H^{•}$ is observed in oxygenated targets until reaching a complete consumption for oxygen concentrations at the water surface larger than 5%. Little impact of the dissolved molecular oxygen has been predicted on the production yield of the other radiolytic species, with the exception of the $H_{2}O_{2}$ whose yield is expected to increase with the target oxygenation especially for high LET radiation tracks. Consistent with the LET dependence of the oxygen effect on the macroscopic level, a strong decrease in the production yields of $O_{2}^{•-}$ and ${\mathrm{HO}}_{2}^{•}$ is observed with the increase of the LET. The strong correlation between the production yields of ($O_{2}^{•-}$ and ${\mathrm{HO}}_{2}^{•}$) and the oxygen radiosensitization effect observed in in vitro cell clonogenic experiments indicate a possible direct or indirect involvement of these species in the indirect radiation damage. Although the code is not yet able to resolve the oxygen effect in biological media and does not account for mechanisms e.g., the oxygen damage fixation, the present implementation provides quantitative insights on the nanoscopic processes involved in the sensitizing effect of oxygen. Further extension of the code to later stages of the chemical dynamics including radiosensitizers, radical scavengers, and specific molecular targets of interest in the cell may be considered.

## Figures and Tables

**Table 2:** List of all the chemical species and their diffusion coefficients, *D*, added to the chemical species list of TRAX to describe the impact of dissolved molecular oxygen in the water target.

	Species	D ($10^{-9}m^{2}s^{-1}$)
	${\mathrm{OH}}^{•}$	2.8
	$H_{3}O^{+}$	9.0
	$H^{•}$	7.0
	$e_{\mathrm{aq}}^{-}$	4.5
	$H_{2}$	4.8
	${\mathrm{OH}}^{-}$	5.0
	$H_{2}O_{2}$	2.3
	$O_{2}$	2.1
	${\mathrm{HO}}_{2}^{•}$	2.0
	${\mathrm{HO}}_{2}^{-}$	2.0
	$O_{2}^{•-}$	2.1
